# A study of prisms and therapy in attention loss after stroke (SPATIAL): A feasibility randomised controlled trial

**DOI:** 10.1177/02692155221134060

**Published:** 2022-10-26

**Authors:** Verity Longley, Kate Woodward-Nutt, Ailie J. Turton, Katie Stocking, Matthew Checketts, Ann Bamford, Emma Douglass, Julie Taylor, Julie Woodley, Pam Moule, Andy Vail, Audrey Bowen

**Affiliations:** 1 Faculty of Health and Education, 5289 Manchester Metropolitan University, Manchester, UK; 2 Geoffrey Jefferson Brain Research Centre, The Manchester Academic Health Science Centre, Northern Care Alliance & University of Manchester, Manchester, UK; 3 School for Health and Social Wellbeing, 1981 University of the West of England, Bristol, UK; 4 Centre for Biostatistics, The Manchester Academic Health Science Centre, Northern Care Alliance & University of Manchester, Manchester, UK; 5 Dorothy House Hospice Care, Bradford-on-Avon, UK

**Keywords:** Stroke, inattention, spatial neglect, rehabilitation, prism, occupational therapy, fidelity, trial, feasibility

## Abstract

**Objective:**

Investigate feasibility and acceptability of prism adaptation training for people with inattention (spatial neglect), early after stroke, during usual care.

**Design:**

Phase II feasibility randomised controlled trial with 3:1 stratified allocation to standard occupational therapy with or without intervention, and nested process evaluation.

**Setting:**

Ten hospital sites providing in-patient stroke services.

**Participants:**

Screened positive for inattention more than one-week post-stroke; informal carers. Occupational therapists participated in qualitative interviews.

**Intervention:**

Adjunctive prism adaptation training at the start of standard occupational therapy sessions for three weeks.

**Main measures:**

Feasibility measures included recruitment and retention rates, intervention fidelity and attrition. Outcomes collected at baseline, 3 weeks and 12 weeks tested measures including Nottingham Extended Activities of Daily Living Scale. Acceptability was explored through qualitative interviews and structured questions.

**Results:**

Eighty (31%) patients were eligible, 57 (71%) consented, 54 randomised (40:13, +1 exclusion) and 39 (74%) completed 12-week outcomes. Treatment fidelity was good: participants received median eight intervention sessions (IQR: 5, 12) lasting 4.7 min (IQR: 4.1, 5.0). All six serious adverse events were unrelated. There was no signal that patients allocated to intervention did better than controls. Twenty five of 35 recruited carers provided outcomes with excellent data completeness. Therapists, patients and carers found prism adaptation training acceptable.

**Conclusions:**

It is feasible and acceptable to conduct a high-quality definitive trial of prism adaptation training within occupational therapy early after stroke in usual care setting, but difficult to justify given no sign of benefit over standard occupational therapy.

**Clinical trial registration:**

https://www.isrctn.com/ Ref ISRCTN88395268.

## Introduction

There is no robust evidence that any therapy reduces the disabling effects of spatial inattention (also known as spatial neglect), a cognitive syndrome affecting awareness towards one side of the body or environment.^1^ National UK audit data from 88,000 hospitalised stroke survivors suggests at least a third screen positive for inattention.^2^ The latter have a longer length of stay and greater dependency at discharge than those screening negative.^3,4,5^ Neglect/inattention (hereafter referred to as inattention as preferred by our patient advisory group) may hinder active participation in rehabilitation.^6,7,8^ An ideal intervention would be an adjunct that, if added at the start of regular occupational therapy sessions, primes the attentional system, enabling patients to engage in therapy.

Prism adaptation training is purported to show short-term relief of spatial deficits but clinical effectiveness has not been evaluated within adequately powered trials.^1^ During prism adaptation, patients point at targets wearing prism glasses which shift their vision laterally. After initially misreaching, they compensate, recalibrating their pointing movements (adaptation). Prisms are then removed and the resulting improvement in cognitive tests and behavioural tasks^9,10,11,12^ can persist for hours.^13^ This paper reports A Study of Prisms And Therapy In Attention Loss after stroke (SPATIAL), a study investigating the feasibility and acceptability of prism adaptation training as an adjunctive intervention early after stroke. Specific objectives were:

1. Determine the feasibility of a future Phase III randomised controlled trial, for example, recruitment and retention of stroke survivors and carers *early* after stroke; ideal setting; value of carer data; whether outcome assessments could be carried by National Health Service (NHS) research support staff; attrition rate; data quality from recruitment and outcome measures; success of outcome assessor blinding; adverse events.
2. Explore the fidelity and acceptability of intervention in the usual care setting.

## Methods

This was a pragmatic, feasibility Phase II multi-centre stratified randomised controlled trial with nested process evaluation, designed with collaborative level patient involvement. The study was approved by the Yorkshire and the Humber NHS Research Ethics Committee (18/YH/048) and entered on the ISRCTN registry, https://www.isrctn.com/ISRCTN88395268.

We monitored trial quality and conduct with a Trial Management Group and an external Trial Steering Committee. ABa, a stroke survivor, worked with the research team in designing the study and was a co-applicant on the grant. ABa identified and chaired a dedicated Patient Carer and Public Involvement advisory group of six stroke survivors, which met ten times during the study. The advisory group provided input on all research activities, from documentation through to dissemination. ABa also represented the group on the trial management group. Two stroke survivors, independent of the advisory group, were members of the trial steering committee.

Participants were recruited between March 2019 and January 2020 with phased site opening. Data collection ended in April 2020. Follow-up assessments took place 3 and 12 weeks after the start of intervention. Reporting follows the CONSORT 2010 statement: extension to randomised pilot and feasibility trials (see Supplemental material).

We recruited participants from NHS in-patient stroke services (provided across 10 acute hospitals and linked rehabilitation facilities) in England. Patients were eligible if they were over 18 years old; had a confirmed stroke (ischaemic or haemorrhagic); positive for spatial inattention at routine screening; had spatial inattention impacting on functional performance; at least one week post-stroke; eligible for standard occupational therapy (for at least one session); able to provide informed consent (or personal/professional consultee available); able to sit with support and perform brief research intervention (e.g. sufficient vision, physical mobility and cognition to be able to participate).

Initially we only included in-patients who were one to four weeks post-stroke but in August 2019 obtained approval for an amendment to widen eligibility beyond four weeks post-stroke. Staff taking consent followed the Mental Capacity Act (2005)^14^ principles and British Psychological Society guidelines when recruiting participants who lacked capacity to consent.^15^ Participants were excluded if they were receiving or expected to receive end of life care; or discharge anticipated before at least one therapy session. Adult informal carers of patient participants were also invited to participate.

NHS occupational therapists identified in-patients with spatial inattention as part of routine clinical care, screening patients for inattention on admission as per each local site practice. NHS research support staff then screened patients for full trial eligibility and gained consent. We used accessible information sheets and consent forms designed with our patient advisory group, alongside consultee declarations for participants deemed unable to give informed consent. NHS research support staff collected standard demographic and clinical data following recruitment including National Institutes of Health Scale (NIHSS). Occupational therapists completed baseline assessments as soon as possible after consent. Occupational therapists also provided a subjective assessment of the severity of patient's inattention on a 4-point scale (none, mild, moderate, severe) using a combination of functional observations and their clinical judgement. Participants no longer displaying inattention at the baseline assessments were withdrawn prior to randomisation.

**Table 1.** Patient participant demographics and baseline clinical data by study arm.

	Intervention; N = 40	Control; N = 13	Whole cohort; N = 53
Gender			
Male n(%)	23 (58%)	7 (54%)	30 (57%)
Female n(%)	17 (43%)	6 (46%)	23 (43%)
Time post-stroke in days (at randomisation)			
Median(IQR)	14 (11, 22)	17 (16, 21)	15 (11, 21)
Min and max	7–77	6–35	6–77
Age at consent			
Mean(SD)	70 (14.3)	67 (9.4)	69 (13.3)
Min-Max	24–89	49–80	24–89
Ethnicity			
White British/Other (%)	39 (98%)	12 (92%)	51 (96%)
Asian/Asian British (%)	1 (3%)	1 (8%)	2 (4%)
Black/African/Caribbean/Black British (%)	0	0	0
Mixed/Multiple ethnic groups (%)	0	0	0
First versus recurrent stroke			
First n(%)	37 (93%)	12 (92%)	49 (92%)
Recurrent n(%)	3 (7%)	1 (8%)	4 (8%)
Type of stroke			
Ischaemic n(%)	30 (75%)	10 (77%)	40 (75%)
Haemorrhagic n(%)	10 (25%)	3 (23%)	13 (25%)
Hemisphere of stroke			
Right n(%)	37 (93%)	11 (85%)	48 (91%)
Left n(%)	3 (8%)	2 (15%)	5 (9%)
Bilateral n(%)	0	0	0
Total NIHSS on admission			
Median (IQR)	14 (8, 17)	11 (4,17)	13 (6, 17)
Min–Max	2–24	0–27	0–27
Missing data n(%)	2 (5%)	1 (8%)	3 (6%)
NIHSS subscore for inattention			
0 n(%)	8 (20%)	6 (46%)	14 (26%)
1 n(%)	15 (38%)	3 (23%)	18 (34%)
2 n(%)	11 (28%)	2 (15%)	13 (25%)
Missing data n(%)	6 (15%)	2 (15%)	8 (15%)
Inattention side			
Left n(%)	37 (93%)	11 (85%)	48 (91%)
Right n(%)	3 (7%)	2 (15%)	5 (9%)
Pre-stroke mRS			
0 n(%)	29 (73%)	6 (46%)	35 (66%)
1 n(%)	6 (15%)	3 (23%)	9 (17%)
2 n(%)	2 (5%)	3 (23%)	5 (9%)
3 n(%)	1 (3%)	1 (8%)	2 (4%)
Missing data n(%)	2 (5%)	-	2 (4%)
Comorbidities			
Congestive HEART failure n(%)	0	0	0
Hypertension n(%)	18 (45%)	3 (23%)	21 (40%)
Diabetes n(%)	4 (10%)	2 (15%)	6 (11%)
TIA n(%)	3 (8%)	1 (8%)	4 (8%)
Atrial fibrillation n(%)	5 (13%)	1 (8%)	6 (11%)
Neurological condition/Brain injury n(%)	2 (5%)	0	2 (4%)
Dementia n(%)	0	0	0
Serious mental health condition n(%)	0	1 (8%)	1 (2%)
Other n(%)	23 (58%)	5 (38%)	28 (53%)
Hearts cancellation			
Number of patients attempted n(%)	36 (90%)	9 (69%)	45 (85%)
Total score median (IQR)	17 (9, 24)	13 (3, 17)	17 (8, 24)
Total score Min–Max	3–47	0–43	0–47
Star cancellation			
Number of patients attempted n(%)	12 (30%)	9 (69%)	21 (39%)
Total score median (IQR)	14 (8, 41)	46 (26, 49)	31 (10, 46)
Total score Min–Max	6–51	2–54	2–54
Reading- number of words/ letters missed			
Median (IQR)	1 (0, 6)	2 (0, 8)	1 (0, 7)
Min–Max	0–14	0–14	0- 14
Missing data n(%)	1 (3%)	-	1 (2%)
KF-NAP total (using actual scores)			
Median (IQR)	18 (11, 23)	16 (10, 18)	16 (11, 23)
Min–Max	2–30	8–27	2–30
Missing data n(%)	1 (3%)	1 (8%)	2 (4%)
Overall clinical impression by occupational therapist			
No inattention	0	0	0
Mild	8 (20%)	4 (31%)	12 (23%)
Moderate	15 (38%)	4 (31%)	19 (36%)
Severe	16 (40%)	5 (38%)	21 (40%)
Missing data n(%)	1 (3%)	-	1 (2%)

NIHSS: National Institutes of Health Stroke Scale; mRS: modified Rankin score; TIA: transient ischaemic attack; KF-NAP: Kessler Foundation Neglect Assessment Process.

We allocated treatment using a 3:1 ratio stratified by site (3 intervention: 1 control), using an independent, web-based, third-party (www.sealedenvelope.com) randomisation service. NHS research support staff performed randomisation following consent and baseline assessment and informed the participant and treating occupational therapist of allocation. Patient participants, treating therapists, the study team and randomising member of NHS research support staff were therefore unblinded to treatment allocation.

For participants in the intervention arm, prism adaptation training was offered once a day at the start of routine occupational therapy sessions, for up to three weeks, five days a week. The training lasted no more than 5 min plus set up time (seating the participants and fitting the glasses). Participants sat at a table in front of a semi-circular board raised approximately 18–26 cm off the table. Participants wore 25 dioptre (12.5°) wedge prism glasses adjusted for left or right sided inattention as appropriate. Occupational therapists or therapy assistants presented a target at the opposite end of the board and asked the participant to reach under the board to touch the target, concealing all but the terminal part of the patient's arm. The therapist presented the target in an unpredictable order and participants pointed to the target for a maximum of 90 movements, or if movements were slow for ≤5 min. The prisms were removed after the pointing task. Details of the prism adaptation session were recorded, including location, delivering staff member, number of movements and length of session.

We trained in-patient occupational therapists and therapy assistants in intervention delivery. We also trained community staff to provide the intervention on discharge if required. A member of the research team was present for the first prism adaptation training session for each participant. See Template for Intervention Description and Replication (TIDieR) checklist (Supplemental material) for additional detail on the intervention.

Following prism removal, participants then received standard occupational therapy. In consultation with therapists, we specified that therapy following prism adaptation should be patient-facing activities in which inattention would affect participation. These were either activities focused on reducing inattention or, for example, functional activities of daily living training in which inattention would affect performance. Non-patient facing therapy activities (e.g. family meetings) were not performed following prism adaptation and staff were asked not to perform prism adaptation prior to standardised or functional assessments where the intervention could impact performance. The treating occupational therapist recorded the frequency, amount and content of each occupational therapy session the participant received during the three-week period using a study-specific data collection form.

The control group received standard occupational therapy (with no prism adaptation training), using the same type of patient-facing activities as the intervention group. Therapy staff recorded the sessions in the same way as the intervention group for the three-week period. In both groups therapy was personalised in line with the National Clinical Guidelines for Stroke,^16^ which suggest patients who need and can tolerate it should accumulate at least 45 min of each appropriate therapy every day. Participants in both groups also received other recommended rehabilitation based on individual need (e.g. physiotherapy, speech and language therapy) as per the Guidelines.^16^ We did not collect data on additional therapies received.

We collected data on candidate outcome measures at three time points. Occupational therapists completed the baseline assessment; NHS research support staff or the study research team collected 3- and 12-week outcomes, either in hospital or at the participant's residence if transferred from in-patient care. The COVID-19 lockdown prevented face-to-face collection of the last few outcome measures however it was possible to collect some by phone. We trained all staff required on baseline and outcome assessment delivery. Outcomes were collected by staff unaware of the patient group where possible.

We collected the following assessments from patient participants at all three timepoints, baseline and 3 and 12 weeks from the start of the intervention:

1. Hearts cancellation test: a subtest of the Oxford Cognitive Screen.^17^ The practice page was given first; if participants had difficulty with the task due to severe inattention or inability to follow assessment instructions the star cancellation was attempted instead.
2. Star cancellation: a subtest of the Behavioural Inattention Test.^18^ Participants completed either hearts *or* star cancellation; star cancellation was used when participants could not complete hearts.
3. Reading test: based on the Radner Reading Test.^19^ Participants read aloud one sentence, printed in size 32 font on an A4 page placed at the participant's midline. We recorded words/letters missed when reading.
4. Kessler Foundation Neglect Assessment Process (KF-NAP)^20^: We asked assessors to follow the standardised instructions, but if unable to assess a particular task we asked for an estimated rating based on their knowledge of the participant.

At 12 weeks only we also collected the:

5. Nottingham Extended Activities of Daily Living scale (NEADL),^21^ the intended primary outcome for a definitive trial
6. Patient Reported Evaluation of Cognitive State (PRECiS),^22^ a patient-centred, patient-reported outcome measure of perception of the impact cognitive problems
7. EQ5D5L^23^
8. Modified Rankin score (mRS),^24^ length of stay and destination on transfer from in-patient care
9. Adverse events up to 12 weeks

We collected the following outcome data from carer participants at 12 weeks only:

1. Carer experience scale^25^
2. Modified carer strain index^26^
3. Self-reported informal carer health service use

We collected data to support the process evaluation through brief structured verbal questions with all patient participants receiving prism adaptation training at 3 weeks post-intervention; and a purposive sample of face-to-face qualitative interviews with seven patient and five carer participants at 12 weeks; and telephone interviews with a purposive sample of 10 occupational therapists following delivery of at least one prism adaptation training session.

Responses to structured questions were coded by PM and JW to identify key phrases and frequency of these across the data. We uploaded transcribed qualitative interview data to NVivo (version 13). Through repeated reading of the transcripts ED and JT familiarised themselves with the data to identify initial codes. Following initial coding, a discussion and comparison was undertaken whereby a consensus was reached, and six themes identified using thematic analysis.^27^

As a Phase II feasibility trial, we did not have a predetermined sample size. Instead, we predicted a recruitment total of 60–80, based on 1–2 participants per month from each site for 12 months (allowing for phased site opening). We reported percentages to assess recruitment, fidelity and attrition. We calculated mean difference and 95% confidence interval for our primary and secondary outcomes on an intention-to-treat basis. We sought outcome data for all participants regardless of treatment adherence unless consent to follow-up was explicitly withdrawn. As there were small numbers of participants, we could not adjust analyses for site (stratification criteria) and baseline severity as planned. Therefore, we used unadjusted regression to obtain the mean difference and 95% CI of the difference for each outcome assessment at T2. We used Stata 14 statistical software.

## Results

**Figure 1.**
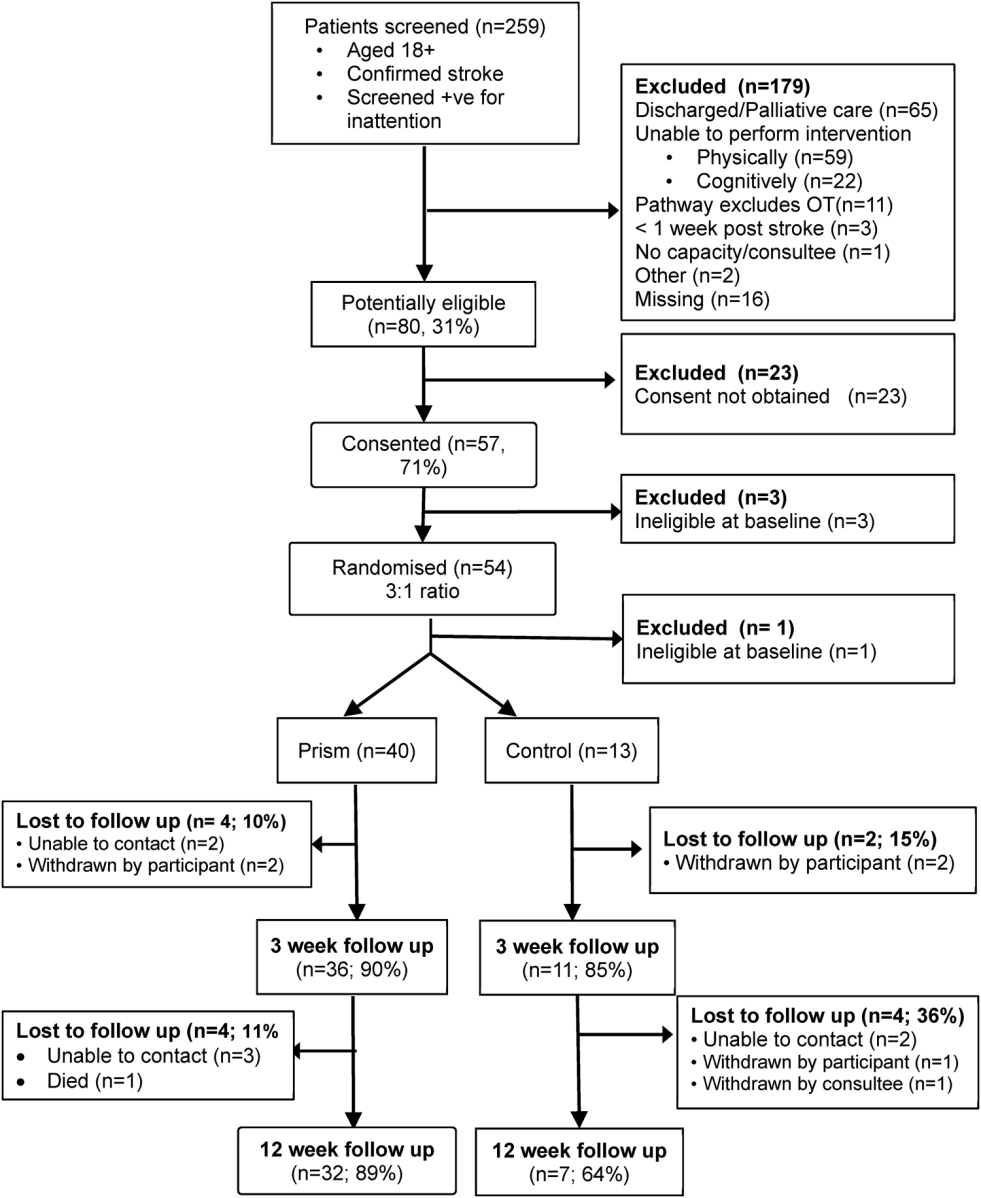
Consort diagram – patient participants.

**Figure 2.**
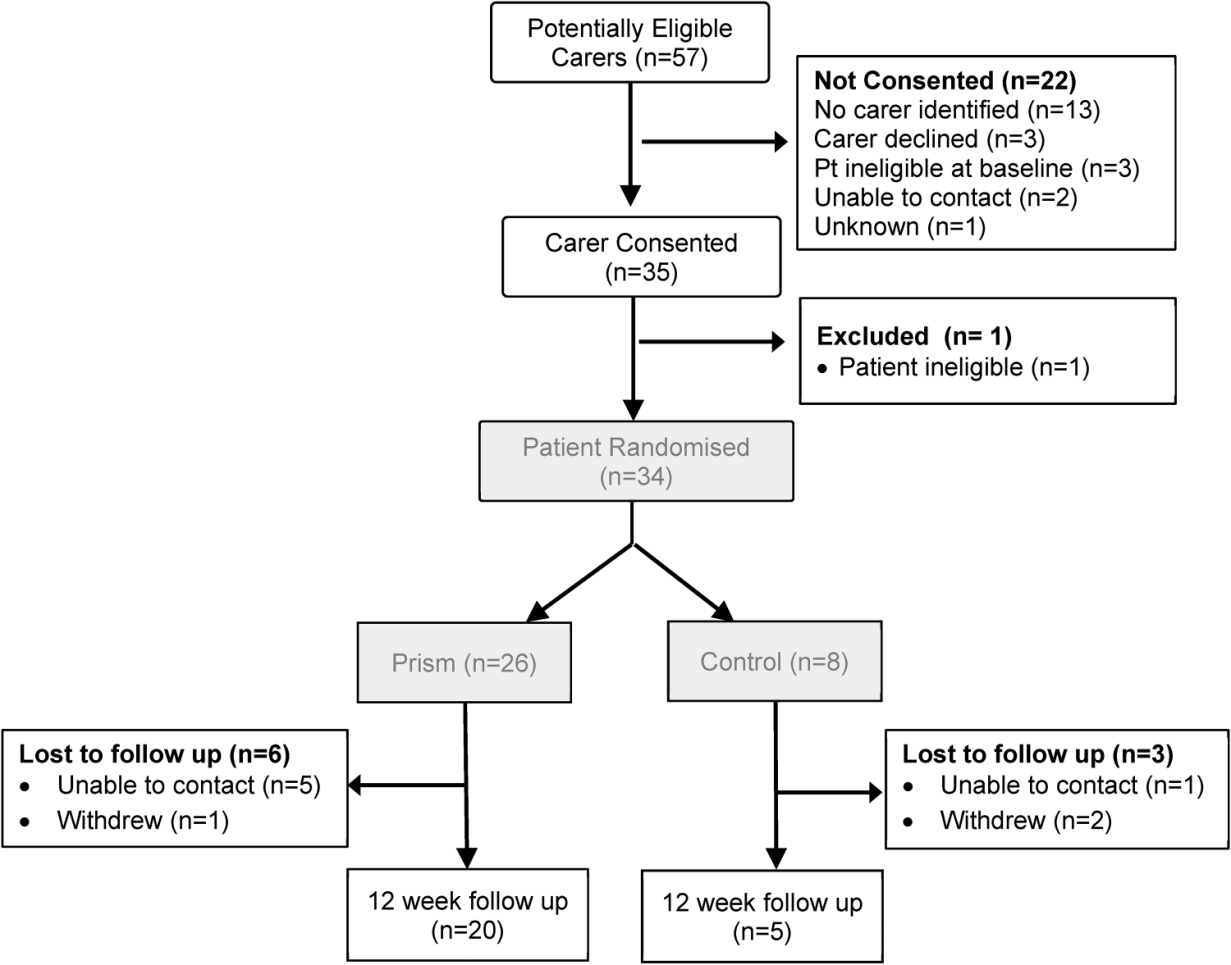
Consort diagram – carer participants.

Figures 1 and 2 show patient and carer participant journey through the 10.5-month recruitment and subsequent follow-up period, including eligibility and consent rates. Recruitment rates varied between sites of different sizes (from 0.2 to 2 patients per site per month and between 0.6% and 4.4% of stroke admissions based on historical admission data).

Thirty-five carers consented to take part in the study. One of these was a carer for a patient who was ineligible at baseline; the carer was therefore also ineligible. Of eligible patient participants, 34 (64%) had an identified and recruited carer (see Figure 2).

Baseline patient participant characteristics are presented in Table 1. The groups were similar in terms of demographic and clinical variables at baseline. The sample had predominantly left inattention (48/53, 91%) and moderate stroke severity on admission with a median NIHSS of 13 (IQR: 6, 17). Participants were recruited from in-patient services a median 15 (IQR: 11, 21) days post-stroke. Two patient participants were discharged prior to the start of the intervention period. Missing data at baseline was minimal, however routinely collected NIHSS sub-score for inattention was missing for eight (15%) patient participants.

Prior to the stroke, eight (24%) carers were not living with the patient participant. Carers were recruited early post-stroke; 13 (38%) were carers of patient participants who had not been discharged to the patient's own home at the 12-week time point. Patient and carer participants generally found recruitment early after stroke acceptable, however some mentioned in the qualitative interviews that deciding to participate was difficult whilst adapting to the stroke. Occupational therapists found the process of identifying participants acceptable and found training beneficial to support study processes.

Prism adaptation training was delivered by occupational therapy staff at all eligible in-patient sites and at three of the six trained community stroke services as part of standard occupational therapy. Two of the three community services provided prism adaptation in participants’ own homes (nine sessions in total) and one provided it (two sessions in total) in a care home where the participant was residing. Prism adaptation training was generally well recorded and delivered as intended: it took a median of 4.7 min (IQR: 4.1, 5.0) and participants made approximately 71.4 (SD 20.0) pointing movements per session. Participants received a median of eight sessions (IQR: 5, 12). Of 322 prism adaptation training sessions offered, only 11 (3%) were declined. Most prism adaptation training sessions took place at the bedside (median 50%, IQR: 13, 77). Minor clinical protocol deviations were that one participant was recorded as having prism adaptation training twice in one day and six participants had it for more than three weeks (see Supplemental material). Of the 40 eligible participants randomised to prism adaptation training there are data for 38 (one withdrawal prior to first intervention, one became unwell).

**Table 2.** Outcome assessment data.

12-week outcome assessment	Intervention	Control	Mean difference, 95% CI
Nottingham Extended Activities of Daily Living scale	N = 32 Mean(SD) = 7.3 (5.2) Min-–Max = 1–18	N = 7 Mean(SD) = 7.9 (6.8) Min–Max = 0–17	−0.6 (−5.3, 4.1)
Kessler Foundation Neglect Assessment Process	N = 31 Median(IQR) = 1.1 (0, 3.8) Min–Max = 0–16	N = 6 Median(IQR) = 2.2 (0, 3.3) Min–Max = 0–5	0.9 (0.2, 4.3)^a^
Patient Reported Evaluation of Cognitive State	N = 30 Median(IQR) = 15 (3, 44) Min–Max = 0–84	N = 6 Median(IQR) = 28 (16, 70) Min–Max = 0–80	2.2 (0.2, 21.0)^a^
Hearts cancellation	N = 29 Mean(SD) = 30.6 (12.3) Min–Max = 7–50	N = 3 Mean(SD) = 47.3 (2.5) Min–Max = 45–50	−16.7 (−1.9, −31.6)
Star cancellation	N = 0 N/A	N = 2 Median(IQR) = 25 (N/A) Min-Max = 13–36	N/A
Reading^b^	N = 30 Zero N(%) = 24 (80%) Non-zero N(%) = 6 (20%)	N = 5 Zero N(%) = 4 (80%) Non-zero N(%) = 1 (20%)	1.0 (0.1,10.7)^b^

a Geometric mean difference and 95% CI for the ratio of means shown for Kessler Foundation Neglect Assessment Process and Patient Reported Evaluation of Cognitive State.

b Reading words missed was dichotomised into zero and non-zero scores and compared using an odds ratio.

We found patient participants generally reported the intervention to be acceptable. In qualitative interviews, patient participants spoke enthusiastically about prism adaptation with a theme of being motivated by an element of personal challenge/competition through repeated intervention sessions. Interviews with occupational therapists confirmed patients found the intervention enjoyable. We identified a theme of perceived benefits of prism adaptation training, with some patients and carers reporting patients were focussing better and having increased awareness following prism adaptation. Seven of 31 participants who completed the structured questions reported finding prism adaptation training tiring, which was also mentioned by one occupational therapist in the qualitative interviews. Both patients and occupational therapists commented on the need for a quiet environment to facilitate concentration on prism adaptation training (see Supplemental material for thematic table).

Standard occupational therapy sessions lasted a mean (SD) of 33.3 (13.1) and 36.4 (12.6) minutes in the intervention and control group, respectively. However, data collection for standard occupational therapy included missing, incomplete and inconsistent data so we are not certain participants in the two arms of the study received equal amounts of therapy time. Sessions were most frequently carried out at the bedside delivered by an individual occupational therapist and included the expected range of therapy activities the most common being ADLs, process training, mobility and upper limb (see Supplemental material).

Attrition was low with three-week outcomes collected from 47 (89%) and 12-week outcomes from 39 (74%) patient participants (see Figure 1). NHS research support staff completed 23 (49%) of the three-week outcomes; and 11/39 (28%) of the 12-week assessments. The University team completed the remaining assessments. Some sites were unable to offer any support with outcome assessments and few were able to assess participants in the community post-discharge.

Forty-one (87%) of 3-week assessments and 31 (79%) at 12 weeks were completed by assessors who recorded that they were unblinded. It was not possible for the NHS research support staff to remain blinded to the study arm as in most instances they were performing the randomisation. Staff were mostly unblinded by the fact that they had been involved in the randomisation process or were members of the University team who had been present at the first prism adaptation training session.

The candidate primary outcome measure, the Nottingham Extended Activities of Daily Living scale at 12 weeks was carried out with all 39 patient participants followed up, with excellent data completion (only missing data for one participant on one of 21 items). Approximately one-third (36%) of participants assessed at 12 weeks were not in their own home (i.e. they were in hospital, rehabilitation unit or care home) and thus not performing many of the extended activities of daily living covered by the measure. Mean differences and confidence intervals on outcome measures are reported in Table 2. See Supplemental material for detailed breakdown of patient and carer outcome assessments.

There were six serious adverse events affecting five participants (four intervention; one control), all of which were assessed by the local Principal Investigators and Chief Investigator as unrelated to the study.

## Discussion

We demonstrated that it would be feasible and acceptable to conduct a definitive trial of a rehabilitation intervention for stroke survivors with inattention, delivered by NHS occupational therapists, beginning very early after stroke in the inpatient usual care rehabilitation setting. We established realistic estimates of recruitment (maximum 2 patients per site per month), retention, data completeness and participant characteristics, and provided data to inform the primary outcome measure and calculate sample size for future trials in this population.

It was not possible for NHS research support staff to complete all outcome assessments, nor for outcome assessors to remain blinded. Attrition in the study was low. Treatment fidelity for prism adaptation training was good in terms of duration and timing of delivery, however recording of the content of occupational therapy sessions was incomplete and needs some revision. Study procedures and prism intervention delivery were acceptable to patients, carers and therapists.

Although the study was not powered to give a conclusion on the effectiveness of prism adaptation training, none of the outcome measures showed any sign of benefit from intervention. Furthermore, a proof-of-concept study (reported separately) did not find evidence to suggest improved engagement in occupational therapy following one session of prism adaptation training. Thus, we cannot justify taking prism adaptation training, provided as per this study and with this early post-stroke population, to a definitive trial.

There are several potential explanations for our findings. There is no standard protocol for prism adaptation delivery, for example, some studies delivered 10 or more sessions per day over two weeks^28^ whilst others used a single session.^29^ Length of session also varies, ranging from 5^30^ to 30 min.^12^ Participants in our study received a median of eight sessions lasting just under 5 min each over three weeks. Other randomised controlled trials using a similar dose found no immediate^31^ nor lasting effects^13^ on cognitive or functional outcomes, however no trial has used it as adjunctive to routine therapy as in this study. It appears that the longer and more frequent the treatment, the more lasting the effects, therefore more intensive treatment may be required for carryover to subsequent therapy sessions.^32,33,34^ This may be impractical in real-world inpatient settings as part of routine therapy.

In addition, time since stroke and physical ability early post-stroke may influence the effect of prism adaptation. Fatigue early post-stroke may mean a higher dose/intensity of prism adaptation is not well tolerated, particularly because people with inattention often have more severe strokes and premorbid difficulties.^2^ Equally, one-third (33%) of patients who screened positive for inattention were ineligible for our study because they were physically unable to carry out the intervention. Participants in studies suggesting effectiveness with intensive treatment were longer post-stroke than our study,^34,35^ therefore level of physical ability may be a limiting factor of usefulness for prism adaptation training in the early stroke population.

Although the sample size of this feasibility study lacks statistical power to identify small but realistic and potentially worthwhile effects, our conclusions are underpinned by our adequately powered proof of concept analysis (reported separately) which ruled out a measurable effect on immediate patient engagement in occupational therapy. The study targeted a potential sample size of 60–80 participants, which resulted in 53 eligible and consenting people. This demonstrated it would be feasible to recruit to a definitive study in the post-acute stage with a slightly longer recruitment window. Whilst our study reflects some existing findings, most existing randomised controlled trials have small samples, many with less than 50 participants, and therefore comparisons should be treated with caution.^30,34,35^

Our choice of outcome assessments may provide limitations. Many participants assessed at 12 weeks were still in hospital, limiting the number of activities of daily living they could report completing on the primary outcome. This also impacted on the relevance of collecting outcome data from carer participants. Despite training, some assessments were not carried out in line with standardised instructions, particularly at baseline (e.g. asking participants to cancel large rather than small stars).

Despite concluding that prism adaptation training, as provided by this study, is not a candidate intervention for a definitive trial, we stress alternative rehabilitation interventions for people with inattention early after stroke are needed. We recommend exploring how patients assessed as physically or cognitively unable to participate in the intervention might be able to benefit from future interventions for inattention. We identified several methodological changes for a future trial, including: choice or timing of most appropriate primary outcome early post-stroke; facilities such as quiet space away from the bedside to facilitate effective intervention delivery; and a need for outcome assessors employed as part of the research team. Blinded assessors are expensive to achieve in rehabilitation trials and trade-offs are required.

**Clinical messages**

- Patients with post-stroke inattention, carers and therapists are willing and able to participate in research early post-stroke in a hospital setting.
- Although brief prism adaptation training was acceptable to deliver at the start of occupational therapy sessions, we did not detect any benefit over and above occupational therapy early after stroke.

## Supplemental Material

sj-doc-1-cre-10.1177_02692155221134060 - Supplemental material for A study of prisms and therapy in attention loss after stroke (SPATIAL): A feasibility randomised controlled trial Click here for additional data file. Supplemental material, sj-doc-1-cre-10.1177_02692155221134060 for A study of prisms and therapy in attention loss after stroke (SPATIAL): A feasibility randomised controlled trial by Verity Longley, Kate Woodward-Nutt, Ailie J. Turton, Katie Stocking, Matthew Checketts, Ann Bamford, Emma Douglass, Julie Taylor, Julie Woodley, Pam Moule, Andy Vail and Audrey Bowen in Clinical Rehabilitation

sj-docx-2-cre-10.1177_02692155221134060 - Supplemental material for A study of prisms and therapy in attention loss after stroke (SPATIAL): A feasibility randomised controlled trial Click here for additional data file. Supplemental material, sj-docx-2-cre-10.1177_02692155221134060 for A study of prisms and therapy in attention loss after stroke (SPATIAL): A feasibility randomised controlled trial by Verity Longley, Kate Woodward-Nutt, Ailie J. Turton, Katie Stocking, Matthew Checketts, Ann Bamford, Emma Douglass, Julie Taylor, Julie Woodley, Pam Moule, Andy Vail and Audrey Bowen in Clinical Rehabilitation

sj-docx-3-cre-10.1177_02692155221134060 - Supplemental material for A study of prisms and therapy in attention loss after stroke (SPATIAL): A feasibility randomised controlled trial Click here for additional data file. Supplemental material, sj-docx-3-cre-10.1177_02692155221134060 for A study of prisms and therapy in attention loss after stroke (SPATIAL): A feasibility randomised controlled trial by Verity Longley, Kate Woodward-Nutt, Ailie J. Turton, Katie Stocking, Matthew Checketts, Ann Bamford, Emma Douglass, Julie Taylor, Julie Woodley, Pam Moule, Andy Vail and Audrey Bowen in Clinical Rehabilitation

sj-docx-4-cre-10.1177_02692155221134060 - Supplemental material for A study of prisms and therapy in attention loss after stroke (SPATIAL): A feasibility randomised controlled trial Click here for additional data file. Supplemental material, sj-docx-4-cre-10.1177_02692155221134060 for A study of prisms and therapy in attention loss after stroke (SPATIAL): A feasibility randomised controlled trial by Verity Longley, Kate Woodward-Nutt, Ailie J. Turton, Katie Stocking, Matthew Checketts, Ann Bamford, Emma Douglass, Julie Taylor, Julie Woodley, Pam Moule, Andy Vail and Audrey Bowen in Clinical Rehabilitation
