# Supplementary material for: A study of prisms and therapy in attention loss after stroke (SPATIAL): A feasibility randomised controlled trial
Source: Clin Rehabil. 2022 Oct 26;37(3):381–93. doi: 10.1177/02692155221134060 (PMC9912302; doi:10.1177/02692155221134060)
Supplement: sj-docx-2-cre-10.1177_02692155221134060 - Supplemental material for A study of prisms and therapy in attention loss after stroke (SPATIAL): A feasibility randomised controlled trial [file sj-docx-2-cre-10.1177_02692155221134060.docx]

**Supplementary material Table 1: Patient baseline clinical outcomes. Kessler Foundation Neglect Assessment Process = KF-NAP.**

|  | **Intervention**  **(N=40)** | **Control**  **(N=13)** | **Whole cohort**  **(N=53)** |
| --- | --- | --- | --- |
| **Hearts cancellation**  Number of patients attempted n(%)  Total Score Median (IQR)  Total Score Min-Max | 36 (90%)  17 (9, 24)  3 – 47 | 9 (69%)  13 (3, 17)  0 – 43 | 45 (85%)  17 (8, 24)  0 – 47 |
| **Hearts cancellation** **space asymmetry**  Left egocentric n(%)  Right egocentric n(%)  N/A (space asymmetry=0) n(%)  Missing data n(%) | 32 (89%)  4 (11%)  0  - | 6 (67%)  1 (11%)  1 (11%)  1 (11%) | 38 (84%)  5 (11%)  1 (2%)  1 (2%) |
| **Hearts cancellation space asymmetry for left neglect patients**  Number with left neglect  Left egocentric n(%)  Right egocentric n(%)  N/A (space asymmetry=0) n(%)  Missing data n(%) | 34  32 (94%)  2 (6%)  0  - | 7  5 (71%)  0  1 (14%)  1 (14%) | 41  37 (90%)  2 (5%)  1 (2%)  1 (2%) |
| **Hearts cancellation object asymmetry**  Left allocentric n(%)  Right allocentric n(%)  N/A (object asymmetry=0) n(%)  Missing data n(%) | 24 (67%)  4 (11%)  8 (22%)  - | 4 (44%)  2 (22%)  2 (22%)  1 (11%) | 28 (62%)  6 (13%)  10 (22%)  1 (11%) |
| **Hearts cancellation** **object asymmetry for left neglect patients**  Number with left neglect  Left allocentric n(%)  Right allocentric n(%)  N/A (object asymmetry=0) n(%) | 34  24 (71%)  2 (6%)  8 (24%)  - | 7  3 (43%)  1 (14%)  2 (29%)  1 (14%) | 41  27 (66%)  3 (7%)  10 (24%)  1 (2%) |
| **Star cancellation**  Number of patients attempted n(%)  Total Score Median (IQR)  Total Score Min-Max | 12 (30%)  14 (8, 41)  6-51 | 9 (69%)  46 (26, 49)  2-54 | 21 (39%)  31 (10, 46)  2 – 54 |
| **Reading- number of words/ letters missed**  Median (IQR)  Min-Max  Missing data n(%) | 1 (0, 6)  0-14  1 (3%) | 2 (0, 8)  0 – 14  - | 1 (0, 7)  0- 14  1 (2%) |
| **KF-NAP total (using actual scores)**  Median (IQR)  Min-Max  Missing data n(%) | 18 (11, 23)  2-30  1 (3%) | 16 (10, 18)  8-27  1 (8%) | 16 (11, 23)  2-30  2 (4%) |
| **KF-NAP neglect severity (actual scores)**  Absent (0) n(%)  Mild (1-10) n(%)  Moderate (11-20) n(%)  Severe (21-30) n(%)  Missing data n(%) | 0  7 (18%)  18 (45%)  14 (35%)  1 (3%) | 0  5 (38%)  5 (38%)  2 (15%)  1 (8%) | 0  12 (23%)  23 (43%)  16 (30%)  2 (4%) |
| **Reading clinical impression**  No Neglect  Mild  Moderate  Severe  Can’t tell  Missing data n(%) | 13 (33%)  6 (15%)  8 (20%)  11 (28%)  2 (5%)  - | 5 (38%)  1 (8%)  3 (23%)  4 (31%)  0  - | 18 (34%)  7 (13%)  11 (21%)  15 (28%)  2 (4%)  - |
| **Hearts cancellation** **clinical impression**  No Neglect  Mild  Moderate  Severe  Can’t tell  Missing data n(%) | 1 (3%)  4 (10%)  12 (30%)  16 (40%)  2 (5%)  5 (13%) | 0  1 (8%)  2 (15%)  5 (38%)  1 (8%)  4 (31%) | 1 (2%)  5 (9%)  14 (26%)  21 (40%)  3 (6%)  9 (17%) |
| **Star cancellation clinical impression**  No Neglect  Mild  Moderate  Severe  Can’t tell  Missing data n(%) | 1 (3%)  1 (3%)  2 (5%)  9 (23%)  1 (3%)  26 (65%) | 1 (8%)  1 (8%)  2 (15%)  2 (15%)  1 (8%)  6 (46%) | 2 (4%)  2 (4%)  4 (8%)  11 (21%)  2 (4%)  32 (60%) |
| **Overall clinical impression**  No Neglect  Mild  Moderate  Severe  Missing data n(%) | 0  8 (20%)  15 (38%)  16 (40%)  1 (3%) | 0  4 (31%)  4 (31%)  5 (38%)  - | 0  12 (23%)  19 (36%)  21 (40%)  1 (2%) |

|  | **Intervention**  **(N=35)** | **Control**  **(N=11)** | **Whole cohort**  **(N=46)** |
| --- | --- | --- | --- |
| **Hearts cancellation**  Number of patients attempted n(%)  Total Score Median (IQR)  Total Score Min-Max | 31 (89%)  30 (11, 36)  4 – 50 | 8 (73%)  34 (13, 43)  3 – 46 | 39 (85%)  30 (11, 37)  3 – 50 |
| **Hearts cancellation space asymmetry**  Left egocentric n(%)  Right egocentric n(%)  N/A (space asymmetry=0) n(%)  Missing data n(%) | 24 (77%)  5 (16%)  2 (6%)  - | 5 (62%)  2 (25%)  1 (13%)  - | 29 (74%)  7 (18%)  1 (13%)  - |
| **Hearts cancellation object asymmetry**  Left allocentric n(%)  Right allocentric n(%)  N/A (object asymmetry=0) n(%) | 19 (61%)  2 (6%)  10 (32%)  - | 4 (50%)  1 (13%)  3 (38%)  - | 23 (59%)  3 (8%)  13 (33%)  - |
| **Star cancellation**  Number of patients attempted n(%)  Total score median (IQR)  Total score min-max | 2 (6%)  24 (N/A)  23-24 | 3 (28%)  39 (N/A)  10-52 | 5 (11%)  24 (23, 39)  10 – 52 |
|  | | | |

**Supplementary material Table 2: Three week outcomes - stars and hearts cancellation tests**

**Supplementary material Table 3: 12 week outcomes - Stars and hearts cancellation tests**

|  | **Intervention**  **(N=32)** | **Control**  **(N=7)** | **Whole cohort**  **(N=39)** |
| --- | --- | --- | --- |
| **Hearts cancellation**  Number of patients attempted n(%)  Total Score Median (IQR)  Total Score Min-Max | 29 (91%)  33 (22, 40)  7 – 50 | 3 (43%)  47 (N/A)  45– 50 | 32 (82%)  35 (24, 41)  7 – 50 |
| **Space asymmetry**  Left egocentric n(%)  Right egocentric n(%)  N/A (space asymmetry=0) n(%)  Missing data n(%) | 18 (62%)  10 (34%)  1 (3%)  - | 1 (33%)  0  2 (67%)  - | 19 (59%)  10 (31%)  3 (9%)  - |
| **Object asymmetry**  Left allocentric n(%)  Right allocentric n(%)  N/A (object asymmetry=0) n(%) | 14 (48%)  4 (14%)  11 (38%)  - | 2 (67%)  0  1 (33%)  - | 16 (50%)  4 (13%)  12 (38%)  - |
| **Star cancellation**  Number of patients attempted n(%)  Total score median (IQR)  Total score min-max | 0  N/A  N/A | 2 (29%)  25 (N/A)  13-36 | 2 (29%)  25 (N/A)  13-36 |
|  | | | |

**Supplementary material table 4: Information on standard occupational therapy (OT) offered**

| **Occupational therapy session information** | **Intervention**  **(N=38)** | **Control**  **(N=13)** | **Whole Cohort**  **(N=51)** |
| --- | --- | --- | --- |
| Average length per session (minutes)  Mean(SD)  Min-Max  Missing data n (%) | 33.3 (13.1)  6.7-65.8  - | 36.4 (12.6)  11.3-59.2  1 (8%) | 34.1 (12.9)  6.7-65.8  1 (2%) |
| Proportion of sessions in each place per person (%):  Bedside - Median(IQR)  Min-Max  Therapy kitchen - Median(IQR)  Min-Max  Bathroom - Median(IQR)  Min-Max  Therapy gym - Median(IQR)  Min-Max  Patient’s home - Median(IQR)  Min-Max  Outside - Median(IQR)  Min-Max  Other therapy area - Median(IQR)  Min-Max | 40 (13,57)  0-100  7 (0, 20)  0-71  10 (0,25)  0-60  11 (0,25)  0-100  0 (0,0)  0-55  0 (0,0)  0-40  4 (0,33)  0-100 | 50 (33,60)  0-100  0 (0,14)  0-100  0 (0,29)  0-42  0 (0,22)  0-67  0 (0,0)  0-14  0 (0,0)  0-22  0 (0,17)  0-29 | 43 (14, 60)  0-100  0 (0,20)  0-100  9 (0,25)  0-60  9 (0,25)  0-100  0 (0,0)  0-55  0 (0,0)  0-40  0 (0,29)  0-100 |
| Proportion of sessions run by (%)  One OT only – Median(IQR)  Min-Max  OT Assistant only - Median(IQR)  Min-Max  >1 member of OT team- Median(IQR)  Min-Max  Joint Session with Other- Median(IQR)  Min-Max | 40 (13,54)  0-88  29 (11, 60)  0-100  0 (0,27)  0-57  8 (0,23)  0-92 | 50 (30,60)  0-100  20 (0,33)  0-100  0 (0,10)  0-20  33 (0,40)  0-58 | 43 (13, 56)  0-100  25 (8,53)  0-100  0 (0,13)  0-57  11 (0,33)  0-92 |
| Proportion of session per person (%)  ADL - Median(IQR)  Min-Max  Process Training - Median(IQR)  Min-Max  Communication - Median(IQR)  Min-Max  Mobility - Median(IQR)  Min-Max  Sensory - Median(IQR)  Min-Max  Upper Limb - Median(IQR)  Min-Max  Group Activity - Median(IQR)  Min-Max  Leisure or Work - Median(IQR)  Min-Max  Outdoor - Median(IQR)  Min-Max  Formal Standardised Function - Median(IQR)  Min-Max | 37 (12,55)  0-100  33 (0,50)  0-100  0 (0,0)  0-100  27 (0,60)  0-100  0 (0,17)  0-100  18 (0,44)  0-100  0 (0,15)  0-100  0 (0,0)  0-100  0 (0,0)  0-27  0 (0,0)  0-100 | 25 (22,50)  0-100  18 (4,67)  0-100  0 (0,16)  0-50  44 (25,50)  0-100  0 (0,25)  0-100  50 (25,63)  0-100  4 (0,35)  0-100  0 (0,4)  0-50  0 (0,0)  0-50  0 (0,5)  0-100 | 36 (14,50)  0-100  27 (0,55)  0-100  0 (0,0)  0-100  30 (0,60)  0-100  0 (0,18)  0-100  25 (0,50)  0-100  0 (0,20)  0-100  0 (0,0)  0-100  0 (0,0)  0-50  0 (0,0)  0-100 |

**Supplementary material table 5: Nottingham Extended Activities of Daily Living Scale (NEADL) at 12 weeks**

| **NEADL domain** | **Whole cohort (n=39)** | | | | |
| --- | --- | --- | --- | --- | --- |
|  | **Not at all** | **With help** | **On own with difficulty** | **On own** | **Missing data** |
| **Walk around outside** | 16 (41%) | 10 (26%) | 4 (10%) | 9 (10%) | - |
| **Climb stairs** | 20 (51%) | 4 (10%) | 4 (10%) | 11 (28%) | - |
| **Get in and out of a car** | 13 (33%) | 11 (28%) | 4 (10%) | 11 (28%) | - |
| **Walk over uneven ground** | 17 (44%) | 9 (23%) | 7 (18%) | 6 (15%) | - |
| **Cross roads** | 21 (54%) | 6 (15%) | 4 (10%) | 8 (21%) | - |
| **Travel on public transport** | 34 (87%) | 2 (5%) | 1 (3%) | 2 (5%) | - |
| **Manage to feed yourself** | 1 (3%) | 5 (13%) | 4 (10%) | 29 (74%) | - |
| **Make yourself a hot drink** | 14 (36%) | 0 | 2 (5%) | 23 (59%) | - |
| **Take hot drinks from one room to another** | 21 (54%) | 1 (3%) | 3 (8%) | 14 (36%) | - |
| **Do the washing up** | 27 (69%) | 1 (3%) | 1 (3%) | 10 (26%) | - |
| **Make yourself a hot snack** | 23 (59%) | 1 (3%) | 2 (5%) | 13 (33%) | - |
| **Manage your own money** | 22 (56%) | 4 (10%) | 2 (5%) | 11 (28%) | - |
| **Wash small items of clothing** | 31 (79%) | 0 | 1 (3%) | 7 (18%) | - |
| **Do your own housework** | 26 (67%) | 0 | 6 (15%) | 7 (18%) | - |
| **Do your own shopping** | 26 (67%) | 7 (18%) | 2 (5%) | 4 (10%) | - |
| **Do a full clothes wash** | 27 (69%) | 1 (3%) | 4 (10%) | 7 (18%) | - |
| **Read newspapers or books** | 14 (36%) | 3 (8%) | 4 (10%) | 18 (46%) | - |
| **Use the telephone** | 11 (28%) | 2 (5%) | 4 (10%) | 22 (56%) | - |
| **Write letters** | 31 (79%) | 1 (3%) | 1 (3%) | 5 (13%) | 1 (3%) |
| **Go out socially** | 23 (59%) | 7 (18%) | 1 (3%) | 8 (21%) | - |
| **Manage your own garden** | 36 (92%) | 1 (3%) | 1 (3%) | 1 (3%) | - |
| **Drive a car** | 37 (95%) | 0 | 0 | 2 (5%) | - |

**Supplementary material table 6: Kessler Foundation Neglect Assessment Process (KF-NAP) breakdown**

| **Domain breakdown (using actual scores)** | **Whole cohort** |
| --- | --- |
| **Gaze Orientation**  **Baseline score**  Number of people at baseline  0 n(%)  1 n(%)  2 n(%)  3 n(%)  Number not scored n(%)  Missing completely n(%)    **Three week score**  Number of people at T1  0 n(%)  1 n(%)  2 n(%)  3 n(%)  Number not scored n(%)  Missing completely n(%)    **12 week Score**  Number of people at T2  0 n(%)  1 n(%)  2 n(%)  3 n(%)  Number not scored n(%)  Missing completely n(%) | 53  3 (6%)  14 (27%)  23 (45%)  11 (22%)  0  2 (4%)  47  22 (48%)  14 (30%)  8 (17%)  2 (4%)  0  1 (2%)  39  26 (70%)  9 (24%)  2 (5%)  0  1 (3%)  2 (5%) |
| **Limb awareness**  **Baseline score**  Number of people at baseline  0 n(%)  1 n(%)  2 n(%)  3 n(%)  Number not scored n(%)  Missing completely n(%)    **Three week score**  Number of people at T1  0 n(%)  1 n(%)  2 n(%)  3 n(%)  Number not scored n(%)  Missing completely n(%)    **12 week score**  Number of people at T2  0 n(%)  1 n(%)  2 n(%)  3 n(%)  Number not scored n(%)  Missing completely n(%) | 53  4 (8%)  17 (33%)  18 (35%)  12 (24%)  0  2 (4%)  47  27 (59%)  15 (33%)  3 (7%)  1 (2%)  0  1 (2%)  39  25 (68%)  10 (27%)  2 (5%)  0  1 (3%)  2 (5%) |
| **Auditory attention**  **Baseline score**  Number of people at baseline  0 n(%)  1 n(%)  2 n(%)  3 n(%)  Number not scored n(%)  Missing completely n(%)    **Three week score**  Number of people at T1  0 n(%)  1 n(%)  2 n(%)  3 n(%)  Number not scored n(%)  Missing completely n(%)    **12 week score**  Number of people at T2  0 n(%)  1 n(%)  2 n(%)  3 n(%)  Number not scored n(%)  Missing completely n(%) | 53  19 (37%)  12 (24%)  18 (35%)  2 (4%)  2 (4%)  2 (4%)  47  28 (61%)  5 (11%)  10 (22%)  3 (7%)  3 (6%)  1 (2%)  39  32 (86%)  1 (3%)  3 (8%)  1 (3%)  1 (3%)  2 (5%) |
| **Personal belongings**  **Baseline score**  Number of people at baseline  0 n(%)  1 n(%)  2 n(%)  3 n(%)  Number not scored n(%)  Missing completely n(%)    **Three week score**  Number of people at T1  0 n(%)  1 n(%)  2 n(%)  3 n(%)  Number not scored n(%)  Missing completely n(%)    **12 week score**  Number of people at T2  0 n(%)  1 n(%)  2 n(%)  3 n(%)  Number not scored n(%)  Missing completely n(%) | 53  4 (25%)  13 (25%)  25 (49%)  9 (18%)  2 (4%)  2 (4%)  47  34 (74%)  6 (13%)  5 (11%)  1 (2%)  3 (6%)  1 (2%)  39  32 (86%)  3 (8%)  2 (5%)  0  2 (5%)  2 (5%) |
| **Dressing**  **Baseline score**  Number of people at baseline  0 n(%)  1 n(%)  2 n(%)  3 n(%)  Number not scored n(%)  Missing completely n(%)    **Three week score**  Number of people at T1  0 n(%)  1 n(%)  2 n(%)  3 n(%)  Number not scored n(%)  Missing completely n(%)    **12 week score**  Number of people at T2  0 n(%)  1 n(%)  2 n(%)  3 n(%)  Number not scored n(%)  Missing completely n(%) | 53  4 (8%)  13 (25%)  21 (41%)  13 (25%)  0  2 (4%)  47  28 (61%)  6 (13%)  12 (26%)  0  4 (9%)  1 (2%)  39  25 (68%)  5 (14%)  7 (19%)  0  6 (15%)  2 (5%) |
| **Grooming**  **Baseline score**  Number of people at baseline  0 n(%)  1 n(%)  2 n(%)  3 n(%)  Number not scored n(%)  Missing completely n(%)    **Three week score**  Number of people at T1  0 n(%)  1 n(%)  2 n(%)  3 n(%)  Number not scored n(%)  Missing completely n(%)    **12 week score**  Number of people at T2  0 n(%)  1 n(%)  2 n(%)  3 n(%)  Number not scored n(%)  Missing completely n(%) | 53  7 (14%)  18 (35%)  20 (39%)  6 (12%)  0  2 (4%)  47  32 (70%)  9 (20%)  5 (11%)  0  1 (2%)  1 (2%)  39  29 (78%)  5 (14%)  3 (8%)  0  2 (5%)  2 (5%) |
| **Navigation**  **Baseline score**  Number of people at baseline  0 n(%)  1 n(%)  2 n(%)  3 n(%)  Number not scored n(%)  Missing completely n(%)    **Three week score**  Number of people at T1  0 n(%)  1 n(%)  2 n(%)  3 n(%)  Number not scored n(%)  Missing completely n(%)    **12 week score**  Number of people at T2  0 n(%)  1 n(%)  2 n(%)  3 n(%)  Number not scored n(%)  Missing completely n(%) | 53  1 (2%)  8 (16%)  29 (58%)  12 (24%)  18 (34%)  3 (6%)  47  23 (50%)  6 (13%)  15 (33%)  2 (4%)  13 (27%)  1 (2%)  39  21 (57%)  3 (8%)  13 (35%)  0  12 (31%)  2 (5%) |
| **Collisions**  **Baseline score**  Number of people at baseline  0 n(%)  1 n(%)  2 n(%)  3 n(%)  Number not scored n(%)  Missing completely n(%)    **Three week score**  Number of people at T1  0 n(%)  1 n(%)  2 n(%)  3 n(%)  Number not scored n(%)  Missing completely n(%)    **12 week score**  Number of people at T2  0 n(%)  1 n(%)  2 n(%)  3 n(%)  Number not scored n(%)  Missing completely n(%) | 53  0  8 (16%)  31 (63%)  10 (20%)  26 (49%)  4 (8%)  47  13 (28%)  5 (11%)  24 (52%)  4 (9%)  13 (27%)  1 (2%)  39  16 (43%)  7 (19%)  14 (38%)  0  12 (31%)  2 (5%) |
| **Meals**  **Baseline score**  Number of people at baseline  0 n(%)  1 n(%)  2 n(%)  3 n(%)  Number not scored n(%)  Missing completely n(%)    **Three week score**  Number of people at T1  0 n(%)  1 n(%)  2 n(%)  3 n(%)  Number not scored n(%)  Missing completely n(%)    **12 week score**  Number of people at T2  0 n(%)  1 n(%)  2 n(%)  3 n(%)  Number not scored n(%)  Missing completely n(%) | 53  5 (10%)  14 (28%)  22 (44%)  9 (18%)  9 (17%)  3 (6%)  47  6 (13%)  3 (7%)  36 (78%)  1 (2%)  34 (72%)  1 (2%)  39  8 (22%)  3 (8%)  26 (70%)  0  25 (64%)  2 (5%) |
| **Cleaning after meals**  **Baseline score**  Number of people at baseline  0 n(%)  1 n(%)  2 n(%)  3 n(%)  Number not scored n(%)  Missing completely n(%)    **Three week score**  Number of people at T1  0 n(%)  1 n(%)  2 n(%)  3 n(%)  Number not scored n(%)  Missing completely n(%)    **12 week score**  Number of people at T2  0 n(%)  1 n(%)  2 n(%)  3 n(%)  Number not scored n(%)  Missing completely n(%) | 53  6 (12%)  15 (30%)  21 (42%)  8 (16%)  8 (15%)  3 (6%)  47  33 (72%)  4 (9%)  9 (20%)  0  7 (15%)  1 (2%)  39  30 (81%)  1 (3%)  6 (16%)  0  6 (15%)  2 (5%) |

**Supplementary material Table 7: Carer outcomes**

|  | **Intervention**  **(n=20)** | **Control**  **(n=5)** | **Overall**  **(n=25)** |
| --- | --- | --- | --- |
| **Carer Experience Scale**  **Activities outside caring**  Can do most other things n(%)  Can do some other thing n(%)  Can do few other things n(%)  Missing completely n(%)    **Support from family and friends**  Get a lot of support n(%)  Get some support n(%)  Get little support n(%)  Missing completely n(%)    **Assistance from organisations and the Government**  Get a lot off assistance n(%)  Get some assistance n(%)  Get little assistance n(%)  Missing completely n(%)  **Fulfilment from caring**  Mostly fulfilling n(%)  Something fulfilling n(%)  Rarely fulfilling n(%)  Missing completely n(%)  **Control over the caring**  In control of most aspects n(%)  In control of some aspects n(%)  In control of few aspects n(%)  Missing completely n(%)  **Getting on with the person you care for**  Mostly get on n(%)  Sometimes get on n(%)  Rarely get on n(%)  Missing completely n(%) | 4 (20%)  4 (20%)  11 (55%)  1 (5%)  10 (50%)  10 (50%)  0  -  2 (10%)  5 (25%)  11 (55%)  2 (10%)  13 (65%)  7 (35%)  0  -  13 (65%)  6 (30%)  1 (5%)  -  18 (90%)  2 (10%)  0  - | 2 (40%)  2 (40%)  1 (20%)  -  1 (20%)  4 (80%)  0  -  0  1 (20%)  4 (80%)  0  2 (40%)  3 (60%)  0  -  1 (20%)  2 (40%)  2 (40%)  -  5 (100%)  0  0  - | 6 (24%)  6 (24%)  12 (48%)  1 (4%)  11 (44%)  14 (56%)  0  -  2 (8%)  6 (24%)  15 (60%)  2 (8%)  15 (60%)  10 (40%)  0  -  14 (56%)  8 (32%)  3 (12%)  -  23 (90%)  2 (8%)  0  - |
| **Modified Caregiver Strain Index**  Mean(SD)  Min-Max  Missing n(%) | 11 (7.1)  0-24  - | 13 (7.1)  4-20  - | 11 (7.0)  0-24  - |
| **Informal Care Costs**  **Number of times in the past month using NHS services for themselves**  Median (IQR)  Min-Max  Missing n(%)  **Have you provided personal care for patient participant?**  Yes n(%)  No n(%)  Missing n(%)  **Have you provided household tasks for patient participant?**  Yes n(%)  No n(%)  Missing n(%)  **Have you provided health tasks for patient participant?**  Yes n(%)  No n(%)  Missing n(%)  **Have you provided other tasks for patient participant?**  Yes n(%)  No n(%)  Missing n(%)  **Estimated hours a day providing care or support**  Mean (SD)  Min-Max  Missing n(%) | 0.5 (0,1.5)  0-9  -  17 (85%)  3 (15%)  -  18 (90%)  2 (10%)  -  16 (80%)  4 (20%)  -  7 (35%)  9 (45%)  4 (20%)  11.2 (8.5)  0.1-24  1 (5%) | 2 (1,7)  0-8  -  5 (100%)  0  -  4 (80%)  1 (20%)  -  4 (80%)  1 (20%)  -  2 (40%)  3 (60%)  -  7.1 (4.7)  4-15  - | 1 (0,2)  0-9  -  22 (88%)  3 (12%)  -  22 (88%)  3 (12%)  -  20 (80%)  5 (20%)  -  9 (36%)  12 (48%)  4 (16%)  10.3 (8.0)  0.1-24  1(4%) |
