# Supplementary material for: A study of prisms and therapy in attention loss after stroke (SPATIAL): A feasibility randomised controlled trial
Source: Clin Rehabil. 2022 Oct 26;37(3):381–93. doi: 10.1177/02692155221134060 (PMC9912302; doi:10.1177/02692155221134060)
Supplement: sj-docx-3-cre-10.1177_02692155221134060 - Supplemental material for A study of prisms and therapy in attention loss after stroke (SPATIAL): A feasibility randomised controlled trial [file sj-docx-3-cre-10.1177_02692155221134060.docx]

**SPATIAL process evaluation table 1: Themes and supporting quotes**

| **Staff training for study** |
| --- |
| “But what I like about this study is we were given quite a lot of information in a folder, so anything we have been told is in there we can go back and flick through.” (OT06)  “It was good to have a go wearing the prisms to see how it felt.” (OT08) |
| **Recruitment** |
| “ …it was all very systematic in terms of how we recruited our patients, we would do that as soon as they were admitted onto the unit… We worked quite closely with the people that were conducting the research via the telephone and also they’d come and visit the unit quite often as well. (OT06)  “One of my biggest concerns about the study has actually already been addressed …if the criteria had extended the timeframe in which, you know the time post stroke that we could have recruited them.” (OT06)  “I said, yes ‘cause I thought it would help other people as well as myself obviously.” (Patient 1)  “I might not have [participated] if it was somebody I didn’t know. (…) It’s always better knowing somebody.” (Patient 3)  “… perhaps I did read [the research documentation], I can’t remember. I probably did read it, but I can’t remember reading it, so…” (Carer 4)  “Yes, I was given information to read and sign that I agreed to everything and it was fine.” (Patient 5) |
| **Timing of PAT** |
| “…I think it’s got to start early in hospital…” (Patient 6)  “I think it can be too early because it's... Particularly if you've never had one [stroke] before. You're angry to what's happening.” (Patient 2)  “So it just came at quite a… (…) It was a bit of a stressful time… (…) Whilst we were happy to do the study, perhaps it could have started a little bit later.” (Carer 3)  “I found it quite useful other than it was a bit tiring doing it with the physical and mental side of it. It's not long since I suffered my stroke so possibly it could be left to maybe a few days longer you might get better results.” (Patient 1).  “It can be quite tiring. I think we have found that people fatigue quite quickly, certainly initially when they are doing it, because then of course, you’ve got your intervention to do afterwards. So, they’ve done a lot of concentrating for that short period of time. So, it can be quite fatiguing.” (OT02) |
| **Motivating aspects of PAT as part of OT sessions** |
| “Yes it was really good I enjoy it because, like I say, it was like having, you had half an hour every day, it was like having company to me, so you were seeing somebody.” (Patient 3)  “That’s what human beings like to do something because all their lives they’re always tested and they want to get good scores and … so they try a bit better…” (Patient 6)  “They’ve all been really engaging and have really enjoyed it. Yes, they’ve found it really good. I’ve had no complaints.” (OT03)  “He’s complaining of pains in his eyes when he’s completing it, and after as well. So, he’s finding that quite uncomfortable, so from the shoulder that’s quite uncomfortable.” (OT02) |
| **Location of PAT** |
| “I thought it was okay in the hospital but it was perhaps…I think with physios coming and meals and everything else, it got a bit hectic at time.” (Patient 1)  “We went to a quiet room which was mainly, probably the kitchen, and we did the training in there.” (Patient 5).  “Well, you’re more relaxed and you’re more in your own environment. I can always say it’s better but obviously if you’ve had it…if it was severe it worked for me in hospital because somewhere it’s got to start.” (Patient 6)  “We’ve got lots of different options in terms of carrying out the intervention. If one room is busy, we’ve always got another option …… on this particular unit we’ve got so many different options in terms of space, we are very very lucky.” (OT06)  “The only thing, is just finding that environment, a suitable environment to do it - that’s quiet enough for those people that are struggling attention-wise, to participate in it. I’m sure that must be challenging everywhere.” (OT01) |
| **Perceived benefits** |
| “Before I’d done the training, I feel I might have struggled, but once I’d done a session of the training and gone out, it was quite good because I felt really confident.” (Patient 5)  “…you could see it happening, you were aware of it happening in your brain. So that few weeks helped me to get over that very severe impairment on my left-hand side (…) eventually it worked because I was getting better with the tests so it was working.” (Patient 6)  “…those Mr Magoo glasses did make a major (…) difference.” (Carer 1)  “… The only thing I might say is I’m not so sure whether it did sort of help me or not. 'Cause I didn’t feel any different having had the tests, so maybe that’s something, I don’t know.” (Patient 1) |
