# Supplementary material for: A study of prisms and therapy in attention loss after stroke (SPATIAL): A feasibility randomised controlled trial
Source: Clin Rehabil. 2022 Oct 26;37(3):381–93. doi: 10.1177/02692155221134060 (PMC9912302; doi:10.1177/02692155221134060)
Supplement: sj-docx-4-cre-10.1177_02692155221134060 - Supplemental material for A study of prisms and therapy in attention loss after stroke (SPATIAL): A feasibility randomised controlled trial [file sj-docx-4-cre-10.1177_02692155221134060.docx]

**SPATIAL Tidier**

| 1. NAME | A feasibility Study of Prisms And Therapy In Attention Loss after stroke: SPATIAL feasibility |
| --- | --- |
| 1. WHY   Describe any rationale, theory, or goal of the elements essential to the intervention | Prism adaptation training (PAT) is a straightforward intervention that aims to remediate spatial inattention by way of visuomotor adaptation.^1^ SPATIAL explores PAT as a means to enable people to participate in recommended NHS Occupational Therapy early after stroke.  PAT is a simple visuo-motor procedure which requires little cognition and so is likely to be able to be used with severely affected stroke patients. However, patients need enough cognitive resources to follow task instructions. Essentially, the participant performs pointing movements to a target for a few minutes while wearing glasses fitted with wedge prisms. The prisms shift the view of the target laterally and initially this causes the participant to miss the targets. However, with repeated pointing the participant adapts and adjusts their pointing to achieve accurate movements. Once the glasses are removed the adaptation has a short-term after-effect in shifting the person’s representation of space further into the neglected side of space.  After prism adaptation patients with spatial inattention have shown long lasting higher order effects with improved performance on neuropsychological tests of spatial attention and some functional tasks. ^2^  It is thought that the treatment triggers a realignment of the egocentric coordinate system that is responsible for the localisation of the body in space and of object position in relation to the body. ^3^ Adding PAT to the beginning of OT sessions might enable people with unilateral spatial inattention to engage in and benefit from recommended OT. |
| 1. MATERIALS   Describe any physical or informational materials used in the intervention, including those provided to participants or used in intervention delivery or in training of intervention providers. Provide information on where the materials can be accessed (e.g. online appendix, URL). | - 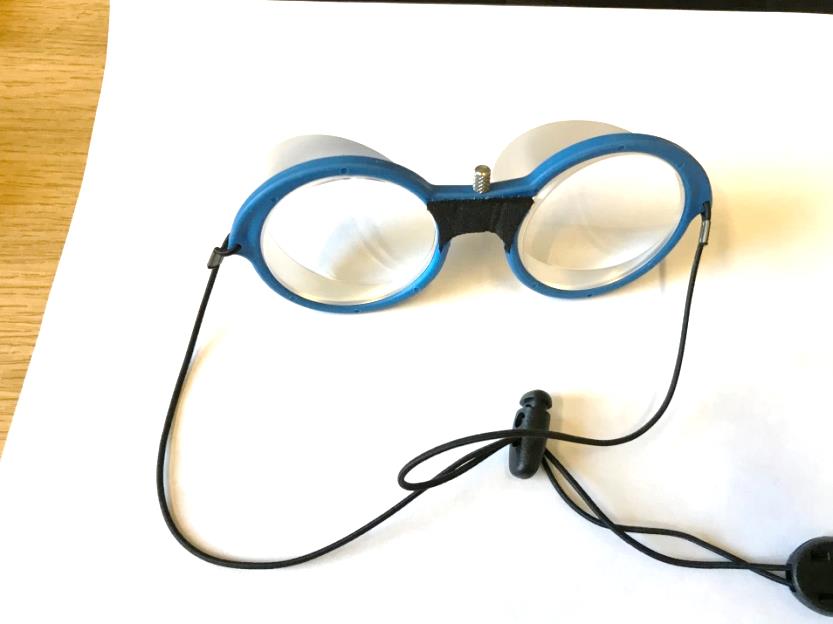Prism glasses: (12.5°, 25 diopter, VTE Vision Training Equipment Stress PoinTest). Prism lenses shift vision laterally by the same amount in each eye. This model of prism glasses allows the prisms to be rotated to have their thick base on the left (for patients with inattention to the left) or to the right (for those with inattention to the right). Once correctly aligned with a mark on the glasses frame a central screw is tightened to keep the lenses in place. Glasses were set up by a member of the research team for each individual participant to ensure that the prisms were correctly orientated. Once set up the glasses were used only for that one participant. - 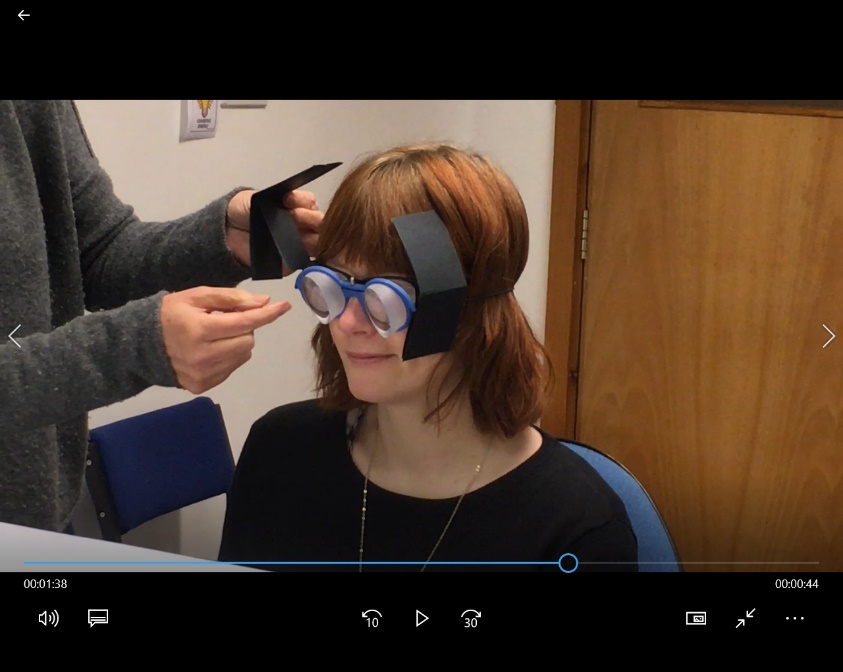Glasses case for individual use for the period of the patient’s intervention. Cases were labelled with participants’ identification number and initials. - Pairs of blinkers for fitting to the prism glasses to prevent conflicting peripheral view. These were made from black card (picture). Additional pairs of blinkers were supplied for each participant. It was recommended that blinkers were replaced weekly, or more frequently as required. - Table and chairs for the patient and therapist to sit opposite one another. Ideally an adjustable height table should be used. The patient may require special seating or supports to ensure optimal upright seated posture. - 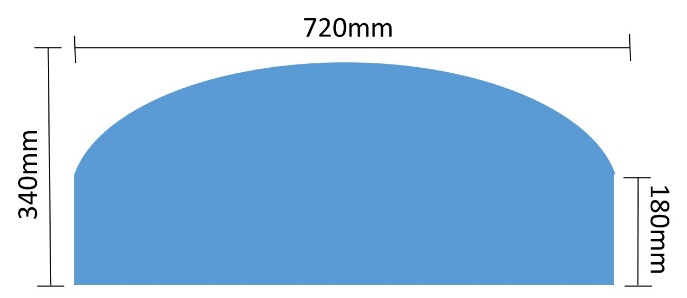Wipe clean A5 box files (260 x 180 x 75mm)) and a loose foamboard lid to create a prism therapy “box”. The lid dimensions: 720mm wide; with depth from 180mm at the sides to 340mm at the centre. The box was designed to fit on a standard hospital cantilever table. The participants are asked to point underneath the lid which is used to occlude the patient’s vision of their pointing trajectory until nearing the target (this is called terminal exposure). All prism therapy equipment is stored inside the box files. - Target (coloured lolly stick) - Log sheet to record session set up, timing and number of pointing movements and use of equipment. - Timer for maximum pointing duration of five minutes. - Clicker counter to record the number of pointing actions (maximum 90). - Post-it notes. A coloured post it note is used to identify the start position of the participant’s unaffected hand. The participant is asked to return their hand to the position after each pointing movement. |
| 1. WHAT PROCEDURES   Describe each of the procedures, activities, and/or processes used in the intervention, including any enabling or support activities. | **Prism Adaptation Training**  Two different methods for administering prism adaptation have been commonly reported in the literature. These differ in the amount of the pointing movement that the participant can see. One method allows the participant to see a large part of the movement as the finger approaches the target and is termed concurrent exposure. The other methods allows only the last few centimetres of the pointing movement to be seen. This is termed terminal exposure. No significant difference between the two methods in effects on either sensorimotor outcomes or neuropsychological outcome measures have been found. ^4^ For the SPATIAL study we used the terminal exposure method. This method was chosen as experience in a previous trial had shown that all participants showed after-effects in their pointing after PAT. ^5^  Before the session materials are collected together and the table is set up with the prism adaptation box and chairs for therapist and for the individual participant, bearing in mind any pillows, specialist seating or equipment that may be needed to support the individual in an upright sitting posture. The participant is seated close up to the table, with the PAT box in front of them. The boxes could be placed in ‘portrait’ or ‘landscape’ orientation to suit the height of the participant relative to the table.  The therapist explains to the participant that they will be required to do repeated pointing movements to a target (lolly stick) on the other side of the box using the hand contralateral to the side of the inattention. They are shown the target and start position (post-it note) on which to place the pointing hand. The start position was, under the box lid, at a comfortable point close to the participant’s body in midline.  Sitting opposite the participant, the therapist shows the participant how they will present the target and that they will be reaching under the screen and that their finger will be visible to them only at the end of the movement as it appears on the other side of the screen. They explain that the participant should return their pointing hand to the start position after touching the target and that the therapist will move the target and then the participant can reach again. A number of practice trials are completed without the prism glasses to make sure the participant understands and can carry out the task and to encourage them to move their arm quickly.  The therapist explains that the pointing will be carried out while wearing prism glasses and shows them the glasses; explaining that the prisms will not affect their ability to see, but that they may find that they have to adjust their pointing a little while they are wearing the prisms for the pointing task. The therapist fits the glasses to the participant and fits the blinkers to the sides so that the participant’s vision is only through the prisms and they cannot see around the edges of them. The therapist checks that the participant can still see the target. For wearers of prescription glasses the prism glasses and blinkers were fitted over the top of the prescription glasses.  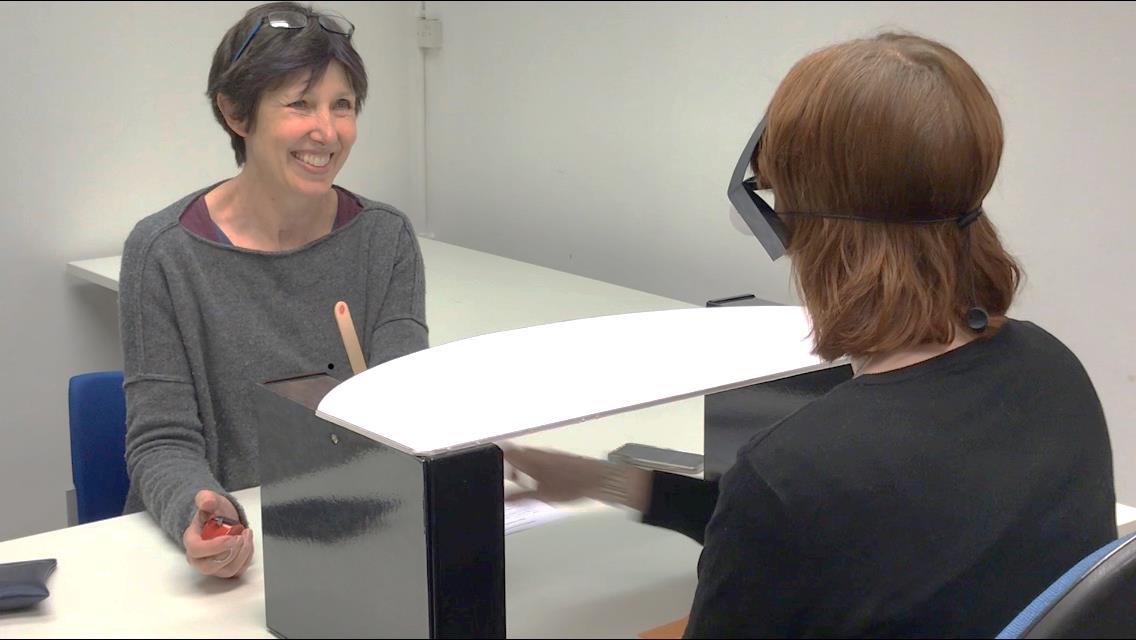The therapist prepares the participant to start the pointing task and tells them that they can rest if they become fatigued. The targets are presented in three different places (centre, left and right) in an unpredictable order. The total number of pointing actions is tallied using the clicker counter. The therapist can prompt the person to scan to the affected side to find the target, or move the target if the participant is unable to see it. Reminders are given to return their hand to the start position and to make the pointing movements to touch the target fairly quickly.  A maximum of 90 pointing movements are carried out, but the task is stopped, and the prism glasses are removed after five minutes even if the 90 movements are not achieved.  The date, time taken and number of pointing movements are recorded on the PAT log sheet.  Immediately after the session the Occupational Therapist carries out standard Occupational Therapy tasks with the participant.  **Occupational Therapy (OT) to follow PAT**  To benefit from the prism adaptation as a primer for Occupational Therapy, we stipulated that the occupational therapy should be active and face to face. We termed the OT for SPATIAL ‘standardised’ OT. The content was produced following consultation with a group of senior occupational therapists. Standardised OT sessions need to have a functional aim. The goal of the session does not have to be focussed on spatial attention. Any therapy activity where the patient’s inattention may affect their participation is relevant. Chosen activities need to involve active attention to the environment or body. For example, the therapy may be aimed at improving upper limb function but using the arm will inevitably require attention to the affected side and to objects in the environment. The following tasks were included:   - - ADL practice e.g. graded kitchen tasks, washing and dressing   - Process training e.g. tabletop activities, visual tasks, reading practice, iPad   - Communication tasks requiring spatial attention e.g. maintaining eye contact in conversation if the patient has difficulty attending to the affected side   - Mobility/transfer practice (including wheelchair)   - Sensory activities e.g. sensory discrimination training   - Upper limb and hand function training e.g. reach and grasp practice   - Group activity e.g. breakfast groups   - Wider community activities e.g. going to shops   - Leisure or work related activities   Formal assessments, including both functional and standardised assessments, were excluded from standardised OT and therapist were asked not to provide PAT prior to assessments to prevent the possibility of PAT influencing the outcome of the assessment. |
| 1. WHO PROVIDES   For each category of intervention provider (e.g. psychologist, nursing assistant), describe their expertise, background and any specific training given. | The SPATIAL intervention, PAT and standardised occupational therapy were provided by Occupational Therapists (216 of 322 sessions) or their support staff (106 of 322 sessions). For practical reasons the intervention did not have to be delivered by the same member of staff at each session.  All occupational therapy staff involved in the study received face to face training on the research process and PAT from one of the research Occupational Therapists. PAT was demonstrated and therapy staff all had an opportunity to trial the prism glasses and practice providing PAT. Staff were all provided with their own study handbook which included detailed PAT instructions with images of PAT set up and PAT being performed. Staff were also provided with a link to a video showing PAT being set up and carried out.  The first PAT session for each participant was attended by a member of the research team who supported therapy staff by responding to queries and provided feedback on how to perform PAT. |
| 1. HOW   Describe the modes of delivery (e.g. face-to-face or by some other mechanism,) of the intervention and whether it was provided individually or in a group. | PAT was carried out face to face with individual participants. Standardised OT was carried out face to face in both one to one and group sessions. |
| 1. WHERE   Describe the type(s) of location(s) where the intervention occurred, including any necessary infrastructure or relevant features. | In the hospital/ rehabilitation unit or in the participant’s home including care home setting. A quiet room was the preferred choice, but often nowhere quiet was available and then PAT was carried out at the bedside.  All participants commenced PAT in hospital/ in-patient rehabilitation unit.  Two participants received some of their PAT sessions at home, and one received PAT in a care home setting. |
| 1. WHEN AND HOW MUCH   Describe the number of times the intervention was delivered and over what period of time including the number of sessions, their schedule, and their duration, intensity or dose. | The aim was to complete PAT once a day, up to 5 times a week for a maximum of 3 weeks. PAT was provided immediately prior to standard OT. When more than one OT session was scheduled in a day the therapist was asked to provide PAT prior to the session most likely to be affected by spatial inattention or at the earlier session if possible.  If participants were discharged during the three-week intervention period PAT could be continued in the community at some sites if PAT trained community staff were available. |
| 1. TAILORING   If the intervention was planned to be personalised, titrated or adapted, then describe what, why, when, and how. | Heights of the table and of the prism adaptation box were adjusted to accommodate the seated height of participants to ensure they were comfortably able to carry out the PAT.  Due to the range of physical, cognitive and visual impairment across the study population, the therapists were advised to personalise some aspects of the intervention. Participants with severe difficulties in maintaining a seated posture were enabled to participate using specialist seating and/or pillows to support them to achieve an upright sitting posture; instructions were modified when necessary so that they could be understood by patients with communication or cognitive problems; participants who struggled to notice the targets were given additional prompts to help them to find it. Therapists were also advised that if the participant was unable to locate the target on the inattentive side then the target could be moved closer to the midline. A median of 54% (IQR: 14, 88) sessions took place with participants seated in a wheelchair. |
| 1. MODIFICATIONS   If the intervention was modified during the course of the study, describe the changes (what, why, when, and how). | No modifications were made to the intervention during the study. |
| 1. HOW WELL   Planned: If intervention adherence or fidelity was assessed, describe how and by whom, and if any strategies were used to maintain or improve fidelity, describe them. | Staff in the study sites were trained initially and were observed at each participant’s first session. They were not observed after that. However the therapy staff were asked to record details of each PAT session on a log sheets to include:   - If blinkers were fitted to the prism glasses - The orientation of the sides of the prism box (landscape or portrait) - How patient was seated (wheelchair/ chair) - If adjustable height table was used - Total number of pointing movements - Length of time for PAT to be completed - If there was a delay of more than 15 minutes before the commencement of the standard OT. |
| 1. Actual: If intervention adherence or fidelity was assessed, describe the extent to which the intervention was delivered as planned. | - Of the 286 PAT sessions completed by 38 participants (median number of sessions 7 IQR 5,10) PAT was typically carried out for up to 5 minutes or a maximum of 90 pointing movements: - There were PAT 21 (7%) sessions, affecting 6 (16%) participants, where more than 90 pointing movements were completed, of which the median number was 103 (IQR: 96, 107) and ranged from 91 to 133. - 2 PAT sessions (< 1%), affecting 2 (5%) participants lasted longer than 5 minutes. Neither was more than 10 seconds beyond the required 5 minutes. - 10 PAT sessions (3%) affecting 6 (16%) of participants did not complete a minimum of 90 pointing movements or 5 minutes of PAT. - 1 (3%) participant had 2 sessions of PAT recorded on one day - 7 (18%) participants received PAT over a period of more than 3 weeks - Blinkers were used with the prism glasses in all but 1 session (<1%) for one participant. |

**References**

1. Rossetti, Y., Rode, G., Pisella, L., Farné, A., Li, L., Boisson, D., & Perenin, M. T. Prism adaptation to a rightward optical deviation rehabilitates left hemispatial neglect. Nature, 1998; 395(6698), 166–169. https://doi.org/10.1038/25988
2. Jacquin-Courtois, S., O’Shea, J., Luaute, J., Pisella, L., Revol, P., Mizuno, K., & Rode, G. Rehabilitation of spatial neglect by prism adaptation. A peculiar expansion of sensorimotor after-effects to spatial cognition. Neuroscience and Biobehavioral Reviews, 2013; 37(4), 594–609. <https://doi.org/http://dx.doi.org/10.1016/j.neubiorev.2013.02.007>
3. Redding, G. M., & Wallace, B. Prism adaptation and unilateral neglect: Review and Analysis. Neuropsychologia, 2006: 44, 1–20.
4. Facchin A, Bultitude JH, Mornati G, Peverelli M, Daini R, A comparison of prism adaptation with terminal versus concurrent exposure on sensorimotor changes and spatial neglect, Neuropsychological Rehabilitation, 2020; 30:4, 613-640, DOI: 10.1080/09602011.2018.1484374
5. Turton AJ, O’Leary K, Gabb J, Woodward R, Gilchrist ID, A single blinded randomized controlled pilot trial of prism adaptation for improving self-care in stroke patients with neglect, Neuropsychological Rehabilitation, 2010; 20:180-96,
